# 5-Hydroxytryptamine, Glutamate, and ATP: Much More Than Neurotransmitters

**DOI:** 10.3389/fcell.2021.667815

**Published:** 2021-04-15

**Authors:** Rafael Franco, Rafael Rivas-Santisteban, Jaume Lillo, Jordi Camps, Gemma Navarro, Irene Reyes-Resina

**Affiliations:** ^1^Department Biochemistry and Molecular Biomedicine, School of Biology, University of Barcelona, Barcelona, Spain; ^2^Centro de Investigación en Red, Enfermedades Neurodegenerativas (CIberNed), Instituto de Salud Carlos III, Madrid, Spain; ^3^Department of Biochemistry and Physiology, School of Pharmacy, University of Barcelona, Barcelona, Spain

**Keywords:** serotonin, adenosine receptors, purinergic signaling, P2 receptors, immune system, heart, COVID-19, inflammatory bowel disease

## Abstract

5-hydroxytryptamine (5-HT) is derived from the essential amino acid L-tryptophan. Although the compound has been studied extensively for its neuronal handling and synaptic actions, serotonin 5-HT receptors can be found extra-synaptically and not only in neurons but in many types of mammalian cells, inside and outside the central nervous system (CNS). In sharp contrast, glutamate (Glu) and ATP are better known as metabolism-related molecules, but they also are neurotransmitters, and their receptors are expressed on almost any type of cell inside and outside the nervous system. Whereas 5-hydroxytryptamine and Glu are key regulators of the immune system, ATP actions are more general. 5-hydroxytryptamine, ATP and Glu act through both G protein-coupled receptors (GPCRs), and ionotropic receptors, i.e., ligand gated ion channels. These are the three examples of neurotransmitters whose actions as holistic regulatory molecules are briefly put into perspective here.

## Introduction

Neurotransmitters like dopamine or serotonin are more known for their actions in the central nervous system (CNS). However, they act both in the CNS and in the periphery as regulators of multiple functions and are key in maintaining whole body homeostasis. Recent studies suggest a dopamine link between the gut and the CNS that is likely mediated by cells of the immune system (see Franco et al., 2021 for review). Lymphocytes and other cells of the myeloid lineage express many receptors for neurotransmitters: dopamine, serotonin, glutamate, etc. It seems that the immune system has evolved in parallel to the nervous system and that the same molecules regulate their functioning. Even the immunological synapse, constituted by the dendritic/antigen-presenting cells and the lymphocyte, is very similar to the canonical synapse established between two neurons or between a neuron and a myocyte (neuromuscular junction). A detailed account of the novel aspects of every neurotransmitter is out of the scope of the present article. We have decided to put into perspective three examples with their analogies and differences, namely 5-hydroxytryptamine (5-HT), glutamate (Glu) and ATP. Glu and ATP share their involvement in cell’s metabolism. 5-HT and Glu share their ability to regulate events of the immune system: from antigen presentation and T-cell activation, to inflammation. 5-HT, Glu and ATP, taken as regulatory molecules, share being the endogenous agonists of two types of cell surface receptors: G protein-coupled receptors (GPCRs) and ionotropic receptors. These examples provide, in our opinion, a perspective on the novel opportunities for better understanding the interrelationships between different organs of the mammalian body and for better knowledge of the physiopathological mechanisms of disease. The extracellular effective concentration of each of these 3 molecules depends on release and uptake, that may vary from tissue to tissue (and from cell to cell). Addressing the mechanisms and proteins involved in release/uptake is out of the scope of the present perspective article. However, it is worth noting that the therapeutic possibilities of receptors for ATP, Glu or 5-HT are limited due to the huge variety of receptors, i.e., by the need of finding highly selective drugs. An alternative approach is to target the transporters. This approach was successful in the case of the Prozac^*R*^ antidepressant drug, that was approved in 1988 and whose active principle, fluoxetine, acts as an inhibitor of 5-HT reuptake, mainly at the level of the presynaptic neuron (Benfield et al., 1986; Fuller, 1986; Sommi et al., 1987).

## Glutamate

**FIGURE 1:**
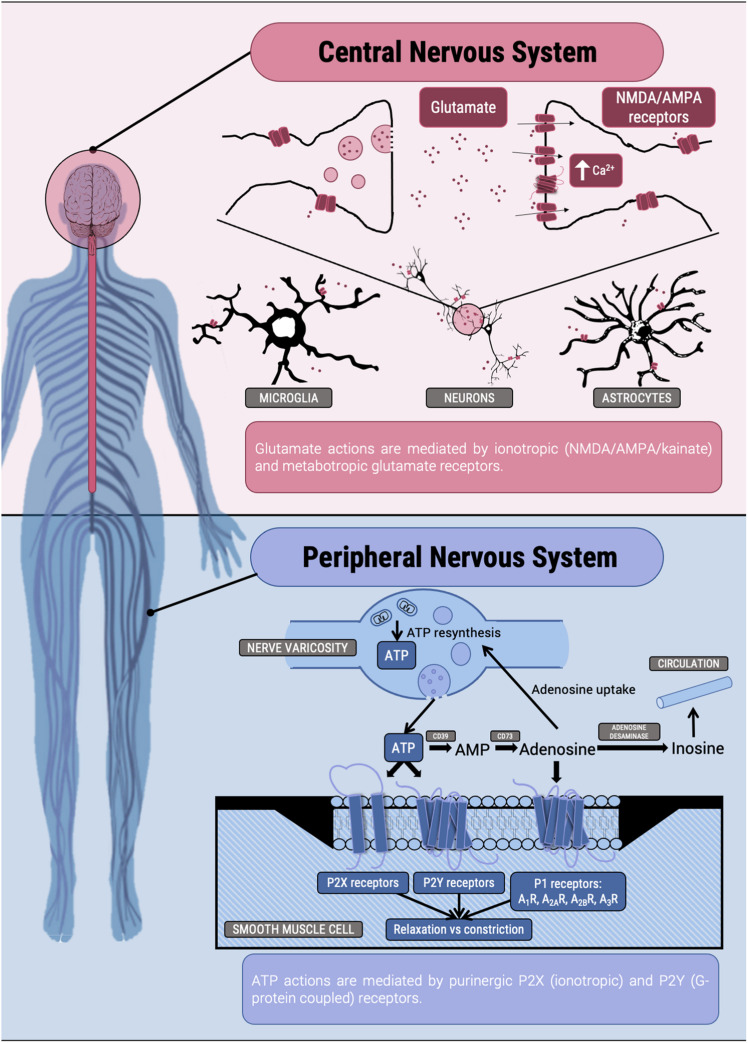
Scheme of some actions of Glu and ATP in the nervous system.

As pointed out by Hertz (2006), acceptance by the neurosciences community of glutamate as the major excitatory transmitter in the CNS did not come until 1984 (Fonnum, 1984; Figure 1). Apart from being one of the 20 amino acids in mammalian proteins, Glu is, directly or indirectly, involved in the metabolism of all cells in the human body. Transamination between Glu and alpha-ketoglutarate is the link to the Krebs cycle, which operates in every cell (erythrocytes are an exception). Its effects as regulator are mediated by metabotropic receptors, which are members of the GPCR superfamily and 3 types of ligand-gated ionotropic channels known as: N-methyl-D-aspartate (NMDA), alpha-amino-3-hydroxy-5-methyl-4-isoxazole propionate (AMPA) and kainate receptors^1^ (Watkins and Jane, 2006). The function as neurotransmitter is mainly mediated by ionotropic receptors that, as metabotropic receptors, are not only expressed in the postsynaptic membrane but also extrasynaptically and in neural cells other than neurons (Borroto-Escuela et al., 2018). Among many other possible examples within the CNS, astrocytes use Glu and glutamine for glia-neuron communication and it is estimated that 20% of cerebral glucose consumption is used to provide energy for Glu metabolism and Glu-mediated actions (Hertz, 2006).

The number of Glu receptors and of receptor combinations is enormous. Ionotropic receptors are constituted by various subunits that form homomers or heteromers. The number of combinations to form functional ligand-gated ion channels is very high as there are, as of today, 16 human genes coding for subunits: 7 of NMDA, 4 of AMPA and 5 of kainate receptors. On top of that, the number of human metabotropic receptors is 8 (mGlu_1_ to mGlu_8_, Alexander et al., 2019), and they can assemble to form homo- or heterodimers, further increasing the number of possible combinations (Duthey et al., 2002; Doumazane et al., 2011; Gregory and Goudet, 2021). Consistent with early regulatory role in Evolution these receptors are not only found in the nervous system but in cells of every any mammalian organ/tissue (O’Rourke and Boeckx, 2020). Glu effects in the periphery are difficult to ascertain due to the doubts on whether the effects are receptor mediated or metabolic. The use of pharmacological tools has, however, circumvented this issue in some cases, for example in the immune system. On the one hand, metabotropic receptors are key modulators of events taking place in the so-called immunological synapse. Dendritic cells release Glu in a non-vesicular manner and using the X_*c*_^–^ cystine/glutamate antiporter. In the absence of antigens the expressed Glu receptors impede activation, whereas after productive antigen presentation, different mGlu receptors are expressed to enhance T cell proliferation and secretion of proinflammatory cytokines (Pacheco et al., 2006). On the other hand, circulating cells of the white lineage express Glu receptors thus showing that they may respond to the amino acid; resting and activated lymphocytes plus human cell lines of lymphoid origin express, at least, mGLu_1_ and mGlu_5_ receptors (Pacheco et al., 2004). It is an open question whether blood Glu levels are important in the control of immune function or it is required a particular environment of cell-to-cell communication in which Glu is released to act locally. Thus, it is unlikely that Glu acts in endocrine fashion. Reviews on Glu role of the physiology of dendritic and T cells are found elsewhere (Pacheco et al., 2007, 2009). Nicoletti et al. (2007) provided an authoritative review on Glu receptor involvement in other non-neural systems and in cancer. In fact, recent work has focused on the facilitation in cancer progression by ionotropic Glu receptors, that arise as targets to manage and refine anti-cancer management (Stepulak et al., 2014; Ribeiro et al., 2017; Ma et al., 2020).

Glu receptor-based therapeutic strategies will require, first, identifying the most convenient target in each disease and, second, the development of highly selective drugs or of allosteric modulators (Nicoletti et al., 2011; Ahmed et al., 2020; Qu et al., 2021).

## ATP

This part of the article is dedicated to the discoverer of purinergic nerves, Prof. Geoffrey Burnstock, who was a fine and inspiring scientist (Hoffmann et al., 2020; Abbracchio, 2021; Franco, 2021). ATP may be released by different cells, including peripheral and central neurons (see Burnstock, 2007 and references therein). Neither identification of ATP as neurotransmitter nor convincing colleagues of this fact (storage in vesicles, release to the synaptic cleft after challenge, etc.) was an easy task. An historical perspective from discovery to the state of the art in 2014 is found in Burnstock (2014). Similar to Glu, ATP is involved in metabolism; actually, ATP is key for life on Earth. Probably for this reason Evolution selected ATP levels as a snitch for homeostasis. The drop in ATP levels would be considered an emergency signal. As for Glu, ATP was first detected as a neurotransmitter to later being found in many systems and is now considered a holistic regulator of whole-body homeostasis. As a neurotransmitter it has a key role in the peripheral nervous system and cumulative evidence points to a key role in visceral pain; Burnstock wrote in 2016 a review that “*describes purinergic mechanosensory transduction involved in visceral, cutaneous, and musculoskeletal nociception and on the roles played by receptor subtypes in neuropathic and inflammatory pain*” (Burnstock, 2016). ATP is also involved in pain derived from cancer via P2X receptors (Wen-Jun et al., 2020; Zhang et al., 2020).

Not as high as for Glu receptors but the number of so-called purinergic receptors, that respond to ATP but also to ADP and pyrimidine nucleotides, is very high. Ionotropic receptors are known as P2X and GPCRs are denoted as P2Y (Figure 1). From X-ray crystallography it is deduced that ionotropic receptors are either homotrimers or heterotrimers, and 7 are the subunits so far discovered (P2X_1_ to P2X_7_). Soon after the cloning of the genes for P2Y receptors and of subunits of P2X receptors, it was clear that extracellular ATP was not only acting as neurotransmitter (Illes et al., 2020; Jacobson et al., 2020). Actually, much of its action is mediated by receptors located in different cells and in different cell locations, e.g., not only in the myocyte membrane part of the neuromuscular junction and not only in smooth muscle cells but also in lymphocytes (see below), the kidney (Vallon et al., 2020), the lung (Wirsching et al., 2020), etc.

As for Glu, and for similar reasons, it is considered that ATP as regulator acts in microenvironments in which close cell-to-cell communication occurs. ATP action in the CNS is important, not necessarily due to neurotransmission mechanisms but via activation by ATP on the multiple P2X and P2Y receptors that are expressed in neural cells. An excellent account of the purinergic action in the brain was probably one of the last papers of Burnstock (2020). Again, the immune system appears as the target of many studies devoted to the role of nucleotides acting via P2X and P2Y receptors. The laboratory of F. di Virgilio has contributed to significantly increase the knowledge on the role of P2 receptors in the control of the immune system function; indeed other laboratories have also contributed (see Rayah et al., 2012; Di Virgilio et al., 2017, and references therein). The P2X_7_ receptor is attracting attention in the last decade for its potential as target in some diseases. Surely, P2X_7_ receptor is involved in regulating infection and inflammation but there is controversy as how to pharmacologically address its potential to make it provide antiinflammatory actions (Di Virgilio et al., 2017; Savio et al., 2018). We would like to add that purinergic signaling is relevant in a prevalent disease for which there are few therapeutic opportunities, namely intestinal inflammation (Longhi et al., 2017).

The section cannot be complete without presenting P1 receptors; there are four (A_1_, A_2A_, A_2B_, and A_3_) all belonging to the GPCR superfamily (Figure 1). Virtually all extracellular ATP is broken down by various nucleotidases to produce adenosine, which is the endogenous agonist of purinergic P1 receptors. Thus, all the regulatory action triggered by ATP is later followed by adenosine receptor-mediated signaling. Adenosine is not considered a neurotransmitter but a neuromodulator. P1 receptors are found in almost any neural cell and also in virtually any peripheral cell in the human body. Importantly, cardiovascular actions of the compound were detected in the twenties (past century) and about 30 years later it was suggested as potential drug to combat tachycardias. Adenosine in bolus administration was approved for the treatment administered with great success to critically ill patients suffering from paroxysmal tachycardia (Drury and Szent-Györgyi, 1929; Wolf and Berne, 1956; Buckley et al., 1959). Further drugs targeting P1 receptors have not been approved as quickly as those targeting adrenergic (beta-blockers) and histamine (H2 antagonists) receptors. P1 receptor antagonist are considered as generally safe. Then natural compounds, caffeine and theophylline, which are non-selective antagonists of these receptors, are approved for human use (Franco, 2009; Franco et al., 2013; Oñatibia-Astibia et al., 2016, 2017), and more recently, an antagonist of the A_2A_ receptor, istradefylline (Mizuno and Kondo, 2013; Kondo et al., 2015), has been approved (in Japan and United States) for adjuvant therapy in Parkinson’s disease. In addition, there are good prospects for P1 receptor ligands to make more effective the immune-based anti-cancer therapy (Ohta and Sitkovsky, 2011; Hatfield and Sitkovsky, 2016; Fong et al., 2020; Sitkovsky, 2020; Willingham et al., 2020).

Finally, we would like to end this section with the very interesting discovery of purinergic (both P1 and P2) receptor involvement in acupuncture-mediated health benefits. To our knowledge the first ATP/acupuncture link was established by Smith and Kenyon (1973) and the first adenosine/acupuncture links were established by Liu et al. (1994a, b). Acupuncture leads to the release of ATP that acts on P2 receptors to produce, among other, analgesia (Burnstock, 2013; Tang et al., 2016, 2019; Tang and Illes, 2020). The subsequent conversion of ATP to adenosine leads to the participation of proximally located P1 receptors (Trento et al., 2021). In summary, there is consensus in that a significant number of acupuncture interventions leads to ATP release to the extracellular milieu and P2 (and P1) engagement.

## Serotonin

5-HT (or serotonin) is known for being one of the main neurotransmitters in the nervous system of mammals; it plays a crucial role as modulator of essential elements of our daily life such as mood, sleep, social behaviors, learning and appetite (Barnes and Sharp, 1999; Gingrich and Hen, 2001; Canli and Lesch, 2007). Although serotonin is best known for regulating higher functions, it is also key in maintaining whole body homeostasis.

While there is a tendency to describe 5-HT as a brain-based and derived molecule, the 95% of the 5-HT is synthetized, stored and released into the gut. This is mainly accomplished by the enterochromaffin cells (Gershon et al., 1965; Costedio et al., 2007; Gershon and Tack, 2007), which are capable of synthetizing it from L-tryptophan by the action of the tryptophan hydroxylase (TPH, Yu et al., 1999). The role of 5-HT in the correct functionality of the gut is a controverted theme, as it is reported that 5-HT modulates gut peristalsis, secretion and motility (Gershon and Tack, 2007), whereas other studies using TPH1 knockout mice, which are unable to synthetize 5-HT, show no alteration of intestinal motility (Li et al., 2011). Nonetheless, it is suggested that serotonin receptors may be targets to combat irritable bowel syndrome (Gershon and Tack, 2007).

There is evidence of a link between the gut and the brain that is somehow mediated by 5-HT (Hooper et al., 2012; Carabotti et al., 2015). This has also been demonstrated for dopamine and it is a hot topic in relationship with Parkinson’s disease (see Franco et al., 2021 for recent review). Among the most likely mechanisms of intercommunication, cells of the immune system may be mediators that respond to 5-HT due to the expression of some of its receptors. It is known that different cell types of the innate immune system, including dendritic cells (O’Connell et al., 2006), monocytes (Finocchiaro et al., 1988) and mastocytes (Tamir et al., 1982) express components of the serotoninergic machinery, e.g., TPH, serotonin transporters and serotonin receptors. Accordingly, it is possible to generate 5-HT and/or to respond to it. A similar trend is found in the adaptive immune system in cells such as T and B lymphocytes (Gordon and Barnes, 2003). Actually, almost any blood cell, except erythrocytes, expresses enzymes related to 5-HT handling and transport, and serotonin receptors. Overall it is assumed that the compound regulates from inflammation to chemotaxis (Pfeiffer et al., 1985; Konig et al., 1994).

5-HT action depends on the cell type and, eventually, in the activation state, for instance in resting versus activated blood cells. On the one hand, 5-HT enhances the production of IFN-γ by human NK cells (Hellstrand et al., 1993) and alters the amount of cytokines released by dendritic cells in such a way that the release of TNF-α and IL-6 is decreased while that of IL-1β and IL-8 is increased (Idzko et al., 2004). On the other hand, in macrophages, 5-HT reduces LPS-induced release of pro-inflammatory cytokines also skewing macrophages to the anti-inflammatory M2 phenotype. The involved receptors are 5-HT_2B_ and 5-HT_7_ (Quintero-Villegas and Valdés-Ferrer, 2019). Furthermore, the use of a 5-HT_7_ receptor agonist in experimental sepsis reduces the plasma levels of IL-6, IL-1β, and of lung NFκB, thus reducing the death rate (Cadirci et al., 2013). However, there are scenarios in which 5-HT potentiates inflammation via other receptors, namely serotonin 5-HT_3_ and 5-HT_4_ receptors (Salaga et al., 2019). The different capacity to modulate cytokine production in opposite outcomes shows that serotoninergic action is receptor and tissue-specific.

Probably the first hints of the regulatory role of 5-HT in inflammation came from evidence of alterations on both immune function and serotoninergic signaling in some psychiatric disorders. Although the 5-HT blood levels have not real diagnostic value, they serve to establish some interesting correlations. Onore et al. (2012) documented alterations in immune function in subjects with autism spectrum disorder that correlated with high blood 5-HT levels (Veenstra-Vanderweele and Blakely, 2012). Correlations between depression and alterations in both immune and serotonergic systems are widely reported in the literature. Levels of pro-inflammatory cytokines such as IL-6 and IFN-γ from T-cells and IL-β1 and TNF-α from the cells of the innate immune system correlate with depression (Raison et al., 2009; Rybka et al., 2016). Also, there is a correlation between risk of depressive moods and single nucleotide polymorphisms found in inflammatory genes crucial for T-cell function. There is even evidence postulating that antidepressant medication can have anti-inflammatory action. For example selective 5-HT reuptake inhibitors, which are used as antidepressants, are reported to reduce and normalize cytokine levels in depressed patients (Basterzi et al., 2005).

SARS-CoV-2 infection leads to COVID-19 that consists of 3 phases that are not manifested in all patients: asymptomatic or low-asymptomatic phase, mildly asymptomatic phase and severe phase (Ayres, 2020). In addition, neurological post-COVID-19 alterations are unexpected side effects of unknown causes and coursing without any defined trend. In psychiatric patients these potential neurological manifestations may aggravate their status. Stressful and anxious conditions may be responsible, at least in part, of post-COVID-19 side effects (Salleh, 2008; Jones and Thomsen, 2013); an increase of IL-6 may also impact in the severity of depression (Zorrilla et al., 2001). In the most severe cases, one of the most harmful events is a robust inflammation with a cytokine release syndrome that may lead to 10-, 100-, and even 1,000-fold increase in the reference blood levels of IL-1β, IL-6, and TNF-α (Chatenoud, 1989). About 40% of the patients treated in the critical care unit display an acute respiratory distress syndrome associated with pneumonia (Guan et al., 2020). In summary, COVID-19 may lead to overstimulation of the immune system (Giamarellos-Bourboulis et al., 2020) and it has been suggested that inhibition of serotonin reuptake may reduce exacerbated inflammation (Hamed and Hagag, 2020). Remarkably, this hypothesis has been supported by a recent study reporting less risk of intubation or death in patients taking antidepressant medication (Hoertel et al., 2021). Inhibitors of 5-HT reuptake could be a novel and effective treatment for severe COVID-19 cases as they could ameliorate the cytokine release syndrome thanks to their capacity to reduce the levels of pro-inflammatory cytokines. For example, these compounds significantly increase oxygen saturation in patients with severe chronic obstructive pulmonary disease (Perna et al., 2004). At the level of speculation, the compounds may act by both reducing inflammation and the levels of anxiety and stress of COVID-19 patients. Data from research with other viruses show that inhibiting serotonin uptake has antiviral properties by downregulating (in lymphocytes) the expression of HIV receptor and coreceptor (Greeson et al., 2016) or by reducing viral replication of Coxsackevirus B4 (Alidjinou et al., 2015). Indeed pancreatic infection with this virus is completely abolished by the widely used 5-HT reuptake inhibitor, fluoxetine (Alidjinou et al., 2015).

Although 5-HT is normally known as a neurotransmitter, it is undeniable that its acts as a holistic modulator in virtually all organs and cell types. It is assumed that every cell in the mammalian body expresses one (or more) of the reported serotonin receptors (14 in total): 5-HT_1A_, 5-HT_1B_, 5-HT_1D_, 5-HT_1E_, 5-HT_1F_, 5-HT_2A_, 5-HT_2B_, 5-HT_2C_, 5-HT_3_, 5-HT_4_, 5-HT_5A_, 5-HT_5B_, 5-HT_6_, and 5-HT_7_ (Barnes et al., 2021). Except the 5-HT_3_, which is an ionotropic receptor, the others belong to the GPCR superfamily; Gi is the canonical G protein of the 1 and 5A types, Gs is the canonical G protein of 4 and 6 receptor types, and members of the 2 type preferentially couple to Gq. There are many unknowns due to the large number of receptors and the potentially different signaling pathways triggered by serotonin receptor activation, but any new scientific achievement will be extremely useful to understand 5-HT action and to identify further serotonin-related targets (transporters or receptors) to combat a variety of diseases.

## Dedication

This article is dedicated to Prof. Geoffrey Burnstock, discoverer or purinergic nerves and founder of a new field of research, who left us in 2020. *Que la tierra te sea leve profesor.*

## Data Availability Statement

The raw data supporting the conclusions of this article will be made available by the authors, without undue reservation.

## Author ContriButions

RR-S, JL, JC, GN, and IR-R compiled information and manuscript from the literature. IR-R and RF did the conceptualization and designed this perspective manuscript. RF and JC wrote the first draft. All authors edited the manuscript and agreed in submitting the final version.

## Conflict of Interest

The authors declare that the research was conducted in the absence of any commercial or financial relationships that could be construed as a potential conflict of interest.

## Footnotes

1 https://www.guidetopharmacology.org/
